# Queuine Micronutrient Deficiency Promotes Warburg Metabolism and Reversal of the Mitochondrial ATP Synthase in Hela Cells

**DOI:** 10.3390/nu12030871

**Published:** 2020-03-24

**Authors:** Patti Hayes, Claire Fergus, Magda Ghanim, Cansu Cirzi, Lyubomyr Burtnyak, Callum J. McGrenaghan, Francesca Tuorto, Derek P. Nolan, Vincent P. Kelly

**Affiliations:** 1 School of Biochemistry & Immunology, Trinity Biomedical Sciences Institute, Trinity College Dublin, 2 Dublin, Ireland; patti.hayes@ucd.ie (P.H.); cfergus@tcd.ie (C.F.); mghanim@tcd.ie (M.G.); burtnyal@tcd.ie (L.B.); mcgrenac@tcd.ie (C.J.M.); denolan@tcd.ie (D.P.N.); 2 Division of Epigenetics, German Cancer Research Center (DKFZ), DKFZ-ZMBH Alliance, 69120 Heidelberg, Germany; c.cirzi@dkfz-heidelberg.de (C.C.); f.tuorto@dkfz-heidelberg.de (F.T.); 3 Faculty of Biosciences, University of Heidelberg, 69120 Heidelberg, Germany; 4 Center for Molecular Biology of Heidelberg University (ZMBH), DKFZ-ZMBH Alliance, 69120 Heidelberg, Germany; 5 Division of Biochemistry, Mannheim Institute for Innate Immunoscience (MI3), Medical Faculty, Heidelberg University, 68167 Mannheim, Germany

**Keywords:** Queuine, queuosine, micronutrient, microbiome, Warburg metabolism, aerobic glycolysis, RNA modification

## Abstract

Queuine is a eukaryotic micronutrient, derived exclusively from eubacteria. It is incorporated into both cytosolic and mitochondrial transfer RNA to generate a queuosine nucleotide at position 34 of the anticodon loop. The transfer RNA of primary tumors has been shown to be hypomodified with respect to queuosine, with decreased levels correlating with disease progression and poor patient survival. Here, we assess the impact of queuine deficiency on mitochondrial bioenergetics and substrate metabolism in HeLa cells. Queuine depletion is shown to promote a Warburg type metabolism, characterized by increased aerobic glycolysis and glutaminolysis, concomitant with increased ammonia and lactate production and elevated levels of lactate dehydrogenase activity but in the absence of significant changes to proliferation. In intact cells, queuine deficiency caused an increased rate of mitochondrial proton leak and a decreased rate of ATP synthesis, correlating with an observed reduction in cellular ATP levels. Data from permeabilized cells demonstrated that the activity of individual complexes of the mitochondrial electron transport chain were not affected by the micronutrient. Notably, in queuine free cells that had been adapted to grow in galactose medium, the re-introduction of glucose permitted the mitochondrial F_1_F_O_-ATP synthase to operate in the reverse direction, acting to hyperpolarize the mitochondrial membrane potential; a commonly observed but poorly understood cancer trait. Together, our data suggest that queuosine hypomodification is a deliberate and advantageous adaptation of cancer cells to facilitate the metabolic switch between oxidative phosphorylation and aerobic glycolysis.

## 1. Introduction

Cancer metabolism is characteristically skewed towards a high rate of glycolysis even in the presence of sufficient oxygen, commonly referred to as aerobic glycolysis or the Warburg effect. The near universality of this phenomenon is evidenced by the clinical success of the glucose analogue [^18^F]-fluorodeoxyglucose to positively diagnose and stage most primary and metastatic lesions [1]. Increased glucose uptake invariably correlates with poor prognosis and aggressive tumor development [2]. However, aerobic glycolysis is only one part of a broader program of cancer transformation which includes rapid glutamine consumption—for anaplerotic replenishment of citric acid cycle intermediates—increased flux through the pentose phosphate pathway, a diminution in oxidative phosphorylation, and increased fatty acid synthesis [3,4]. This metabolic switch is generally understood to arise from altered activity or expression of oncogenes (e.g., myc, AKT, Her2), tumor suppressor genes (e.g., p53), and regulators of the hypoxic response (HIF1α) [5].

**Figure 1:**
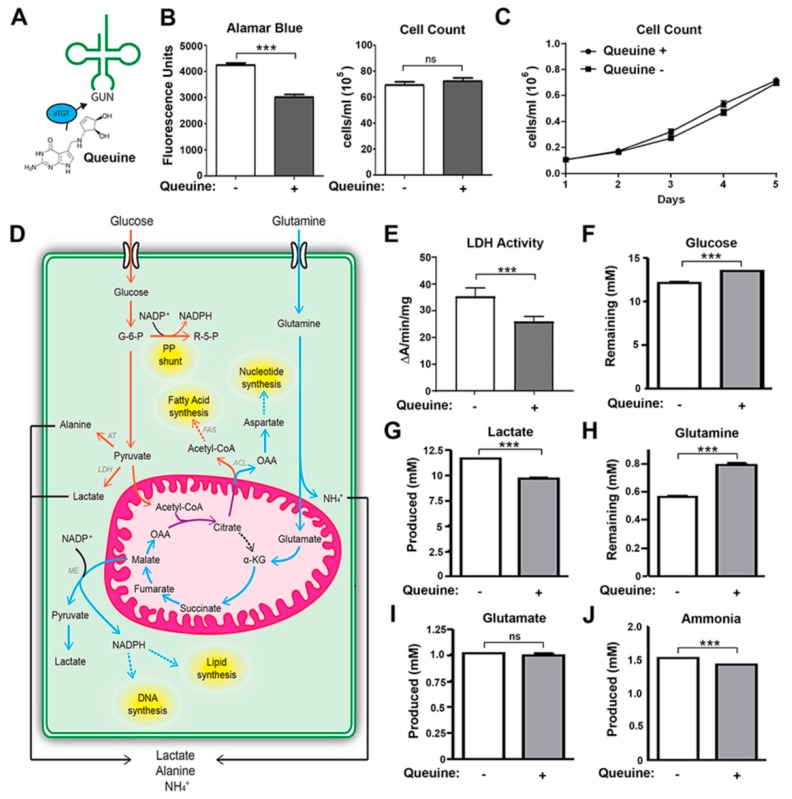
HeLa cells deficient in queuine experience a Warburg type metabolism. (**A**) The eukaryotic TGT (eTGT) enzyme irreversibly replaces guanine with queuine base in the wobble position of nuclear and mitochondrial tRNA containing a G_34_U_35_N_36_ anticodon (where N is any nucleotide). (**B**) Cells were seeded at 3 × 10^4^ per cm^2^ in serum free medium (queuine deficient) for 5 days or supplemented with 1 µM queuine base every 24 h. Viability and cell number were determined by Alamar blue assay and manual counting on a hemocytometer, respectively. (**C**) Queuine deficiency does not lead to changes in the rate of HeLa cell proliferation. Cells were grown as described above and counted daily using a hemocytometer. (**D**) Warburg metabolism is characterized by increased aerobic glycolysis. Glucose and glutamine supply the majority of carbon, nitrogen, free energy, and reducing equivalents required for cancer cell growth and division, with the concomitant secretion of lactate, alanine, and ammonia. Glucose may be redirected towards the pentose phosphate (PP) shunt for NADPH and ribose production. Acetyl-CoA from glucose and oxaloacetate (OAA) from glutamine converge to produce citrate, which is exported to the cytoplasm and converted to fatty acids through the action of the ATP-citrate lyase (ACL) and fatty acid synthase (FAS). Oxaloacetate may also be converted into aspartate to support nucleotide synthesis. Malate is exported to the cytoplasm where it is converted to lactate by malic enzyme (ME) and in the process produces NADPH necessary for the synthesis of lipids and DNA. Pyruvate is converted to lactate by the enzyme lactate dehydrogenase (LDH) and to alanine by alanine transaminase (AT), which is secreted from the cell. (**E**) HeLa cells were cultured for 48 h in serum-free medium in the absence (white bar) or presence (gray bar) of 1 µM queuine. Total protein was extracted and LDH activity determined per mg of protein. (**F–J**) Cells were grown in serum free medium without further additions (white bar) or supplemented daily (gray bar) with 1 µM queuine. At 72 h, the culture medium was filtered and the indicated substrates and metabolites quantitated using a Nova Bioprofile 400 analyzer. Mean (± s.d.) for triplicate samples. *n* ≥ 2. ***p < 0.001, *t*-test.

Queuine is an oft-overlooked micronutrient of both unicellular and multicellular eukaryotic species including algae, fungi, plants, and humans. It is exclusively recovered from eubacterial species that are uniquely equipped for its biosynthesis [6]. Following cellular uptake, queuine is inserted into the anticodon loop of transfer RNA (tRNA) containing a GUN anticodon sequence (tRNA_GUN_; where N is any nucleotide) representing the tRNA isoacceptors for tyrosine, histidine, asparagine, and aspartic acid [6,7]. The eukaryotic enzyme that performs this reaction, tRNA guanine transglycosylase (eTGT), catalyses a base-for-base exchange reaction, wherein the nuclear encoded guanine at position 34 (the wobble base) is removed and replaced with the queuine micronutrient, generating a queuosine nucleotide or Q-modification (Figure 1A). In addition to being present in cytosolic tRNA_GUN_, direct experimental evidence demonstrates that the Q-modification is present on mitochondrial tRNA. This has been shown for tRNA^Asp^ from rat [8] and opossum [9] and for the four bovine mitochondrial tRNA_GUN_ species [10].

Intriguingly, from the perspective of a metabolite derived from bacteria, studies have revealed a marked depletion of Q-modified tRNA_GUN_ in neoplastic samples from solid and blood-borne cancers. Specifically, Q-hypomodification has been shown in the case of ovarian cancer [11], lung cancer [12], leukaemia, lymphoma [13,14], astrocytoma, and meningioma [15]. Further, the progression from benign to metastatic lesions was found to correlate with decreasing levels of Q-modified tRNA, which has been suggested as a possible predictor of poor long-term survival [11,12,14].

Evidence from cell lines provides a somewhat fragmentary explanation for the effect(s) of Q-depletion in cancer. The Kersten group reported that queuine supplementation of growth medium has a cell-type specific effect on proliferation, being either positive (HeLaS3, A-431, HL-60) or negative (Colo-DM320, PC-12, EAT) [16,17]. An association with oncogene levels and oncogene driven growth has also been reported. The proliferation of NIH-3T3 cells was found to be positively affected by queuine but repressed when cells are transformed with the oncogenes *ras, erbB2* or *raf* [18]. In HeLaS3 cells, queuine addition was shown to reduce *c-fos* transcript abundance and to increase those of *c-myc* [16]. Links with metabolism have also been described. Under hypoxic conditions, the addition of queuine to the culture medium was found to decrease HeLaS3 cell proliferation and conversely to increase growth under aerobic conditions. In the same study, queuine deficiency was found to increase lactate dehydrogenase (LDH) A4 levels under aerobic conditions, an effect that could be reversed by addition of queuine [17]. Transplantation of Dalton’s lymphoma ascites into mice was found to increase LDH-A isozyme levels in the serum and liver, which was reversed by queuine administration [19]. Finally, a demonstrable interrelationship between queuine and cellular differentiation has been observed. In the K562 (human) and 745A (murine) erythroleukemia cell lines, Q-tRNA levels increase with differentiation status [20,21]. Trewyn and colleagues have shown that transformation of C3H10T1/2 murine fibroblasts with oncogenic *ras* leads to queuine depletion and enhanced anchorage independent growth [22], while research from the same group showed that an inhibitor of eTGT, 7-methylguanine, can increase anchorage independent growth and serve as a tumor promoter in a two-stage initiation-promoter cancer model [23,24]. Conversely, the replacement of queuine with 6-thioguanine in the tRNA of human promyelocytic HL-60 cells was found to promote cellular differentiation [25,26,27,28].

The research to date raises the tantalizing possibility that Q-hypomodification in cancer is a deliberate, rather than a passive, event and may confer selective advantage to the growing tumor. In this study, we sought to isolate the metabolic and proliferative effect of queuine on HeLa cells using defined serum-free conditions. Furthermore, as a key driver of metabolism and the transformation process, the impact of Q-hypomodification on mitochondrial function and activity was determined in both intact and permeabilized cells.

## 2. Materials and Methods 

### 2.1. Hela Cell Culture

HeLa cells (ECACC, 93021013) were maintained in Eagles minimal essential medium (EMEM) supplemented with 2 mM L-glutamine, 0.1 mg/mL penicillin-streptomycin, and 10% FBS. Queuine deficiency was induced by growth in Ultraculture™ serum-free medium (Lonza Group, Basel, Switzerland) supplemented with 2 mM L-glutamine and 0.1 mg/mL penicillin-streptomycin for at least three passages. Galactose medium (10 mM) was prepared from custom synthesized Ultraculture medium deficient in glucose. Queuine, a kind gift from Dr. Susumu Nishimura, was prepared as a stock solution (100 μM) in ultrapure H_2_O and added to cells at the concentration and times shown. Cell viability was assessed using alamarBLUE^®^ (ThermoFisher Scientific, Waltham, Massachusetts, United States) according to manufacturer’s instructions.

### 2.2. Detection of Q-modified tRNA Using Acryloyl Aminophenylboronic Acid Gels 

Acryloyl aminophenylboronic acid (APB) gels were prepared and run as previously described by Zaborske et al. [29]. Briefly, RNA was deacetylated and denatured prior to loading on APB gels. Gels were stained with SYBR Gold nucleic acid stain solution. Northern blotting was performed in a semi-dry system. The RNAs were blotted on a positively charged nylon membrane (Version 19, Roche) in 1x TAE buffer at 5 volts for 45 min. To cross-link the RNAs with the membrane, UV light was applied twice for 1200 s. The membrane was added to hybridization buffer containing 5x SSC (0.3 M trisodium citrate, 3M NaCl, pH 7.5, 20 mM Na_2_HPO_4_ pH 7.2, 7% SDS, 2x Denhardt’s solution) at 42 °C for 1 h. Labeled probe (γ-^32^P) was applied to the membrane in hybridization buffer and incubated at 42 °C overnight on a rotator. The membrane was washed twice for 15 min at 42 °C in 2x SSC, 5% SDS solution and for 15 min at room temperature with 1x SSC, 1% SDS solution before being exposed to autoradiographic film. 

### 2.3. Enzyme Activity Assays

Lactate dehydrogenase (LDH) activity was measured from the oxidation of NADH at 340 nm. Reactions were initiated by the addition of 20 µg total protein from cultured cells to 200 mM Tris-HCl, pH 7.3, 6.6 mM NADH, and 30 mM sodium pyruvate. Citrate synthase (CS) activity was measured using a coupled reaction, wherein the CoASH product is reacted with 5,5-dithio-bis-nitrobenzoic acid (DTNB) to yield 5-thionitrobenzene, a colored compound whose absorbance can be measured at 412 nm. The assay was carried out in acrylic cuvettes containing 0.1 M Tris-HCl, pH 8.1, 0.15 M sucrose. The absorbance was set to zero before the addition of 50 µl DTNB (4 mg/mL), 50 µl acetyl CoA (2 mM), and 20 µg total protein from cultured cells. The contents of the cuvette were agitated for 3-4 min. The reaction was initiated by the addition of 75 µl oxaloacetate (60 mM). The DTNB, acetyl CoA, and oxaloacetate were all freshly prepared. Cytochrome c oxidase activity was determined by measuring the rate of oxidation of reduced cytochrome c at 550 nm. Cytochrome c (Sigma Aldrich, Arklow, Co. Wicklow, Ireland) was reduced by the addition of several crystals of ascorbic acid (Honeywell Fluka, Charlotte, North Carolina, NC, USA) until a color transition from red to brown was observed. The excess ascorbic acid was removed by passing the reduced cytochrome c through a PD10 gel filtration column (GE healthcare) equilibrated with 10 mM potassium phosphate buffer, pH 7.0. The concentration of the reduced cytochrome c was determined spectrophotometrically using cytochrome c oxidized with 1 M potassium ferricyanide as a blank. The cytochrome c oxidase reaction was carried out in 10 mM potassium phosphate buffer and 10 µM reduced cytochrome c. Background rate was observed for 1 min before addition of 200 µg protein and initial rates were used to calculate activity.

### 2.4. ATP Measurement

CellTiter-Glo^®^ (Promega, Madison, WI, USA) is a luminescent reagent used to quantitate ATP levels in metabolically active cells. Cells were seeded with or without queuine for 48 h. On the day of the experiment, the plate was equilibrated at room temperature for 30 min before an equal volume of CellTiter-Glo Reagent was added to each well. The plate was placed on an orbital shaker for 2 min to induce lysis. After standing the plate for 10 min at room temperature, luminescence was recorded.

### 2.5. Metabolite Analysis

A Nova Bioprofile 400 Analyzer (Nova Biomedical, MA, USA) was used to measure the levels of glucose, lactate, glutamine, glutamate, and ammonia in the culture medium. Cells were seeded at a density of 7 × 10^4^ cells/cm^2^ in medium lacking sodium bicarbonate and cultivated with no medium change for 72 h. Medium was removed and passed through a 0.22 µm filter before analysis on the Nova Bioprofiler.

### 2.6. Mitochondrial and Nuclear DNA Quantitation

Mitochondrial and nuclear DNA copy number were evaluated by quantitative PCR (qPCR) using SYBR^®^ Green dye on an Applied Biosystems 7500 system. The PCR was performed for 35 cycles (10 s at 98 °C, 30 s at 60 °C, and 30 s at 72 °C) using the mitochondrial COX1 forward (5′-CGT TGA CTA TTC TCT ACA AAC CAC-3′) and reverse (5′-GAA GAT TAT TAC AAA TGC ATG GGC-3′) primers and the nuclear NDUFV1 forward (5′-CTT CCC CAC TGG CCT CAA G-3′) and reverse (5′-CTG GGA ACA TGT GCC CAC CTC -3′) primers to generate amplicons of 189 bp and 176 bp, respectively.

### 2.7. Mitochondrial Membrane Potential Analysis

Radiolabeled triphenylmethylphosphonium bromide (TPMP) was used to estimate mitochondrial membrane potential. The equilibrium distribution of this lipophilic cation is a direct measure of membrane potentials according to the Nernst equation. Cells (1 × 10^7^) were resuspended in DMEM lacking FBS, antibiotics, or phenol-red but containing [^3^H]TPMP^+^ (5 nM, 0.1 µCi/mL) and 5 nM tetraphenylboron (TPB; a lipophilic anion that aids equilibration of TPMP^+^ into the cell) for 80 min. In parallel experiments, the energy dependency of TPMP^+^ accumulation was determined by incubations supplemented with 12 µM oligomycin or 20 µM carbonyl cyanide-p-trifluoro-methoxphenylhydrazone (FCCP), prior to addition of TPMP^+^/TPB. Cells were layered on top of 100 µl of diisooctylphthalate:diisobutylphthalate (1:2) before being spun at 10,000 × g at 4 °C for 30 s and 100 µl of the supernatant collected. The remainder of the supernatant and oil layer was carefully removed by aspiration and the pellet was resuspended in 100 µl 20% Triton X-100 (v/v) and 1 mL of Ecoscint scintillation cocktail was added to both supernatant and pellet before the samples were counted on a scintillation counter. The energization dependent TPMP^+^ uptake was expressed as an accumulation ratio calculated using the equation (TPMP^+^/mg protein)/(TPMP^+^/μL supernatant) [30,31]. 

### 2.8. Transmission Electron Microscopy

A total of 1-2 × 10^7^ HeLa cells were fixed with 4% glutaraldehyde solution in situ for 1 h. Cells were scraped off the surface of the dish and washed twice with 50 mM phosphate buffer. An equal volume of 2% agarose was added to the cell pellet and allowed to solidify at 4 °C for 30 min before being cut into approximately 4 mm^2^ slices. These slices were washed for 10 min in 50 mM phosphate before 2% osmium tetroxide (OsO_4_) in 50 mM phosphate was added. Cells were incubated for 45 min before the OsO_4_ was aspirated off and the samples dehydrated using a series of alcohol solutions of increasing percentage concentration (30–95% aqueous ethanol solutions at 10 min intervals). Samples were then held in 100% ethanol overnight before being washed twice for 15 min each with 100% propylene oxide at room temperature. An equal volume of epoxy resin was then added to the propylene oxide suspended slices and allowed to incubate at room temperature for 3 h on a rotor. The 50% epoxy resin solution was replaced with a 100% solution and allowed to incubate on a rotor at room temperature for a further 3 h. Slices were then removed from the resin solution before being placed in a mould and covered with fresh 100% resin. The mould was placed in an oven set at 50 °C and degassed for 1 h before the temperature was increased to 60 °C and left overnight. Ultrathin sections were cut on an ultramicrotome and collected on copper grids. Each grid was counterstained with uranyl acetate and lead citrate before being examined using a JEOL 1210 electron microscope.

### 2.9. Mitochondrial Function Analysis

An Oroboros Oxygraph-2K was used for respirometric analysis. The Oroboros was calibrated with respiration medium MiR05 (500 µM EGTA, 3 mM MgCl_2_.6H_2_O, 60 mM K-lactobionate, 20 mM taurine, 10 mM KH_2_PO_4_, 20 mM HEPES, 110 mM sucrose, 1g/L fatty acid free BSA, adjusted to pH 7.1 with KOH) at 37 °C for approximately 1 h. The medium was stirred at 540 rpm until calibration at air saturation was attained, as evidenced by a stable oxygen flux. Cells were introduced into the 2x cm^3^ chamber at a density of 2 × 10^6^ cells/mL or greater. Respiratory control analysis was carried out on intact cells. Once a basal rate of respiration had been established, oligomycin was added at a concentration of 1 µg using a Hamilton microsyringe. After a stable rate of respiration was observed for five minutes, FCCP was added to give a 3 µM final concentration (pre-determined to give full uncoupling). Uncoupling was verified by the further addition of 500 nM FCCP, which failed to induce any further increase in oxygen consumption. The addition of rotenone and antimycin A at 0.5 µM and 2.5 µM (final) was used to inhibit respiration so that the contribution of non-oxidative side reactions to the rate of oxygen consumption could be ascertained. To examine the activity of respiratory chain complexes, HeLa cells were permeabilized with 0.005% (w/v) digitonin, the concentration of which was pre-optimized to ensure continued mitochondrial viability. The basal rate of respiration was observed to decrease before stabilizing due to dilution of substrates. Glutamate (10 mM) and malate (5 mM) were introduced to the chamber to provide NADH for complex I (state 2 respiration), followed by saturating concentrations (2 mM) of ADP (state 3 respiration). Complex I was inhibited with 500 nM rotenone before succinate (10 mM) was added to support complex II respiration (state 3). Complex II was inhibited with malonate (10 mM) and glycerol-3-phosphate added (10 mM) to support complex III respiration. Inhibition of complex III with antimycin A (5 µM) was followed by stimulation of complex IV with 100 µM TMPD and 400 µM ascorbate. Complex IV was inhibited by the addition of potassium cyanide (700 µM). In set-up assays, after the above process, the integrity of the mitochondrial membrane was checked by the addition of cytochrome c (10 µM) and was found to result in little or no increase in the rate of respiration. The ATP/O ratio was determined using permeabilized cells. Adenosine kinase activity was inhibited through the addition of 75 µM of P1, P5-Di(adenosine-5′) pentaphosphate and once a stable rate of respiration had been established, the NADH-linked substrates glutamate (10 mM) and malate (5 mM) were added, followed by 50 µM ADP. The rate of oxygen consumption was calculated from the oxygraph trace.

### 2.10. Confocal Microscopy

Cells were washed with pre-warmed growth medium before the addition of Mitotracker Red CMXRos (50 nM) or Mitotracker Green FM dye (40 nM) in non-quench mode, along with 0.2 μg/mL DAPI for 15 min at 37 °C. Cells were washed with pre-warmed medium before fixing in 4% w/v paraformaldehyde. Cells were analyzed on an Olympus IX81 Fluorescent microscope.

## 3. Results

### 3.1. Queuine Deficiency Induces Aerobic Glycolysis and Glutaminolysis in Hela Cells 

In previous studies, the effect of queuine on cell proliferation was examined using horse serum or dialyzed fetal bovine serum, which have low to undetectable amounts of the micronutrient [16,18,32]. However, to avoid any possible compounding effects of the culture medium, in this study HeLa cells were grown in defined serum-free conditions (Ultraculture medium), previously determined to be queuine free [33]. Control and experimental samples differed exclusively by the absence or presence of chemically synthesized queuine base at a concentration of 1 µM which was found to fully modify tRNA within 24 h (Figure S1). In initial experiments, the fluorometric growth indicator Alamar blue was used to quantitate viable cell number after 48 h in culture. A robust increase in fluorescence was observed in the absence of queuine (Figure 1B), however manual cell counts did not mirror the changes seen, questioning the reliability of the Alamar blue approach to evaluate cell number; vide infra. Therefore, cells were manually counted each day for five days using a hemocytometer. There was no statistically significant difference in the proliferation rate of cells grown in the absence or presence of queuine (Figure 1C). 

Glucose and glutamine are two of the principle carbon sources for proliferating cancer cells and provide the bioenergetic needs and metabolic intermediates required for macromolecular synthesis (Figure 1D). The metabolic change is coupled to an elevation of LDH that facilitates the higher rate of aerobic glycolysis [34]. The observation that LDH isoforms may be influenced by queuine [18,19] led us to examine LDH activity in lysates from HeLa cells grown for 48 h in the absence or presence of queuine. Queuine deficient (and hence Q-hypomodified) cells had approximately 25% greater levels of LDH activity relative to queuine-supplemented cells (Figure 1E). The metabolism of glucose and glutamine leads to a concomitant cellular production of lactate, glutamate, and ammonia Therefore, in addition to the precursor substrates we examined the levels of each of these key metabolites in the culture medium of HeLa cells grown in the absence or presence of queuine for 72 h (Figure 1F–J). Clear increases in glucose and glutamine consumption coupled to an increase in ammonia and lactate release were observed in queuine deficient cells relative to those supplemented with queuine. 

The above data indicate that in the absence of the queuine micronutrient, HeLa cells adopt an enhanced level of cancer-like metabolism (Warburg effect). This metabolic bias has numerous potential underpinnings in the context of tRNA or possibly from other unknown actions of the queuine base itself. Our original observation that the addition of queuine to cells caused a marked decrease in Alamar Blue fluorescence lead us to question whether a deficiency in queuine may be affecting mitochondrial respiration. Alamar blue is known to act as an intermediate electron acceptor for the electron transport chain [35], in addition to other mitochondrial reductases and oxidoreductase enzymes [36]. It should be noted that, in contrast to our own observations, previous reports using HeLaS3 cells showed that the intracellular reduction of the tetrazolium dye MTT was increased 1.4 fold in the presence of queuine, which was interpreted to result from increased mitochondrial electron flow [17].

### 3.2. Queuine Deficiency Affects Mitochondrial Membrane Hyperpolarisation by Oligomycin

The genome of human mitochondria encodes 13 proteins that are essential subunits of the electron transport chain (ETC) and 2 subunits of the F_1_F_O_-ATPase, in addition to 22 mitochondrial tRNAs and 2 ribosomal RNAs. Conceivably, queuine depletion and the absence of queuosine in mitochondrial tRNA_GUN_ could limit mitochondrial translation efficiency that in turn could affect mitochondrial metabolic function resulting in an increased level of aerobic glycolysis. This putative impairment could manifest in changes in mitochondrial number, morphology, or defects in the activity of individual components of the ETC and the F_1_F_O_-ATPase, or a combined effect of the above.

**Figure 2:**
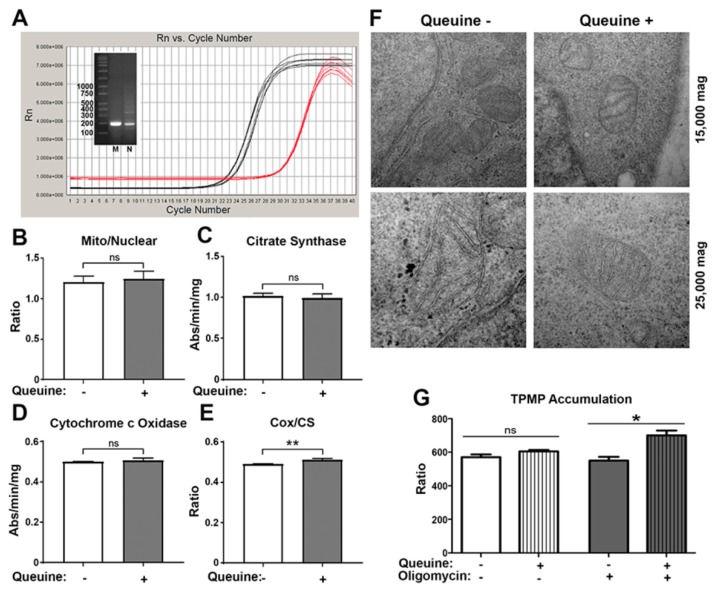
Mitochondrial membrane potential is affected by queuine deficiency. HeLa cells were cultured for 48 h in the absence or presence of 1 µM queuine, as indicated. (**A**–**B**) Total DNA was extracted and examined by quantitative PCR (upper panel) for the mitochondrial encoded cox1 gene (black trace) and the nuclear encoded ndufv1 gene (red trace) producing amplicons of 189 bp and 176 bp respectively (inset figure; M, mitochondrial; N, nuclear) and the ratio of mitochondrial number to nuclear DNA was determined. (**C–E**) Protein extracts were examined for citrate synthase and cytochrome c oxidase activity, and the ratio of both values calculated to provide an index of ETC activity to mitochondrial volume. (**F**) Following fixation, dehydration, and embedding, HeLa cells were sectioned and counterstained with uranyl acetate and lead citrate for analysis by TEM (JOEL 1210 microscope) at 15,000× magnification (top row) and 25,000× magnification (bottom row). (**G**) Mitochondrial membrane potential was examined by the uptake of the lipophilic cation TPMP^+^. Energy dependent TPMP^+^ uptake, in the absence (left) or the presence (right) of the F_1_F_O_ ATP synthase (F_O_-subunit) inhibitor oligomycin, was expressed as the accumulation ratio per mg of protein relative to TPMP^+^ concentration in the medium according to the equation (TPMP.mg^-1^ protein/TPMP.µL^-1^ supernatant). Mean (± s.d.) for triplicate samples. *n* ≥ 2. *p < 0.05, **p < 0.01, *t*-test.

Quantitation of mitochondrial number and activity was made using DNA- and protein-based methods from cells cultured for 48 h in the absence or presence of queuine. Determination of mitochondrial number using real-time PCR (Figure 2A)—wherein the quantity of the mitochondrial encoded *COX1* gene was compared to that of the nuclear encoded *NDUFV1* gene—showed no change with queuine (Figure 2B). Citrate synthase (CS) is a soluble mitochondrial matrix protein whose amount correlates with mitochondrial number [37]. Its activity was determined indirectly using a coupled spectrophotometric assay to measure the production of reduced acetyl-CoA (Figure 2C). The membrane bound cytochrome c oxidase (Cox) activity was also measured as a gauge of oxidative phosphorylation (Figure 2D) and the ratio of these enzyme activities (Cox/CS) calculated to provide an index of ETC activity relative to mitochondrial volume (Figure 2E). Although the individual activity of these enzymes was not visibly affected by the absence of queuine, a statistically significant decrease in the Cox/CS ratio (*p* < 0.01) was observed relative to queuine-supplemented conditions, although the effects were marginal. The data may hint that the mitochondrial capacity to engage in oxidative phosphorylation is compromised in the absence of queuine; however it is doubtful the moderate changes seen would impact overall ETC function. 

Mitochondria may also modulate their shape in accordance with bioenergetic needs [38]. For example, cells grown in the presence of galactose—the oxidation of which provides no net production of ATP—results in an extended and branched mitochondrial network together with increases in membrane cristae and matrix density [39]. The morphology of cells grown with and without queuine was examined by electron microscopy, however no discernible differences in matrix volume or cristae number could be observed (Figure 2F).

The mitochondrial membrane potential represents the major component of the proton-motive force for ATP production and is directly required for the import of proteins and substrates into the mitochondria and therefore its evaluation (in addition to respiration below) is highly informative of mitochondrial function. The membrane potential was measured using the distribution at equilibrium of the lipophilic cation methyltriphenylphosphonium (TPMP^+^). This probe crosses biological membranes by passive diffusion and accumulates in response to membrane potential. Total cellular accumulation of TPMP^+^ by eukaryotes is a product of the plasma and mitochondrial membrane potentials since both are negative [40]. Cells were cultured in the absence or presence of queuine for 48 h before the accumulation of TPMP^+^ was determined (Figure 2G). There was no significant difference in the total accumulation of TPMP^+^ by cells grown in the absence or presence of queuine (left), which was consistent with the view that both the plasma and mitochondrial membrane potentials were of similar magnitude in both cases. Interestingly, in queuine treated cells there was increased accumulation of TPMP^+^ following the addition of oligomycin (i.e., increased membrane potential), a cell permeable F_1_F_O_-ATP synthase inhibitor (right). Curiously however, in queuine deficient cells the oligomycin treatment did not show a correspondingly similar increase in membrane potential. This may be explained by a reduced rate of substrate oxidation or increased proton leak by mitochondria in queuine deficient cells.

### 3.3. Queuine Deficient Cells Display an Increased Proton Leak and Reduced Rate of ATP Synthesis

**Figure 3:**
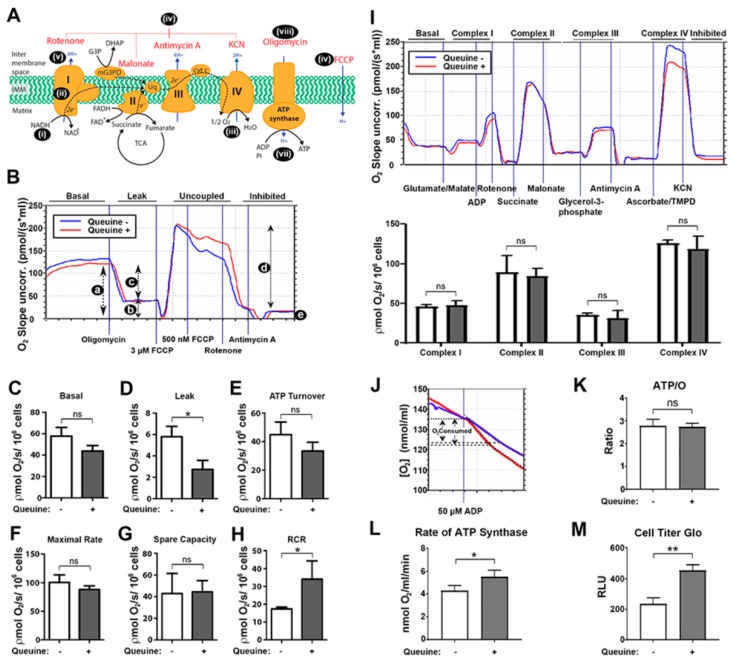
Electron transport chain (ETC) activity is normal in queuine deficiency whereas leak and ATP synthesis are affected. (**A**) Mechanism and inhibition of the electron transport chain (ETC). The ETC (**i**) acquires free energy from food substrates (glucose, amino acids, fatty acids) through the intermediacy of NADH cofactor, succinate, and glycerol-3-phosphate (also fatty acids; not shown). Electrons flow stepwise (**ii**) through a chain of carrier complexes I-IV, situated in the mitochondrial inner membrane, with oxygen (O_2_) acting as the terminal electron acceptor (**iii**), which is reduced to water. Electron flow can be interrupted at each complex using specific inhibitors (**iv**), as shown. Electron movement along the ETC is coupled to proton translocation from the mitochondrial matrix into the intermembrane space (**v**) creating a protomotive force across the inner membrane, which can be artificially dissipated by the ionophore FCCP (**vi**). The flow of protons back across the membrane through the ATP synthase (complex V) drives ATP synthesis (**vii**) from ADP and inorganic phosphate (Pi). The activity of the ATP synthase in both forward and reverse directions can be inhibited by oligomycin (viii). (**B**) HeLa cells were cultured in the absence (blue lines) or presence (red lines) of 1 µM queuine for 48 h, harvested, resuspended in MiR05 buffer, and 4 × 10^6^ cells were introduced into each chamber of an Oroboros oxygraph-2K instrument. The parameters a. basal respiration, b. oligomycin-insensitive respiration (leak; state 4o), c. oligomycin-sensitive (ATP turnover) respiration, d. maximum respiratory capacity in the presence of FCCP (state 3u), and e. the non-mitochondrial respiration rate were determined (representative trace) by the addition of the indicated agents (top figure). (**C**) Basal, (**D**) leak, (**E**) ATP turnover, (**F**) maximal/uncoupled rate, (**G**) spare capacity, and (**H**) respiratory control ratio (RCR) are graphed from repeat experiments. (**I**) Respirometric analysis of the ETC. After endogenous respiration had stabilized, glutamate and malate were added as substrate for complex I, cells were permeabilized with digitonin and respiration stimulated with the addition of ADP (upper panel; representative trace). Subsequently, the indicated substrates and inhibitors were added to examine the activity of complexes I–IV. Data from repeat experiments were graphed (lower panel). (**J**) The ATP/O ratio was measured using the NADH-linked substrates glutamate and malate (representative trace). Cells were permeabilized with digitonin and adenylate kinase inhibitor Ap5A added to each chamber. Once a stable rate of respiration was established a fixed concentration of ADP (50 µM) was added, the O_2_ consumption recorded and the ATP/O ratio (**K**) and rate of ATP synthesis (**L**) calculated. (**M**) The level of cellular ATP was measured using CellTiter-Glo reagent. Graphed data represent mean (± s.d.) for triplicate samples. *n* = 3. *p < 0.05, **p < 0.01, ns, not-significant, *t*-test.

Given the differences seen above in the membrane potential under queuine deficient conditions, an evaluation of mitochondrial respiration was made using intact cells (as opposed to isolated mitochondria). This provides a physiologically relevant measurement of mitochondrial function supported by endogenously supplied substrates. The F_1_F_O_-ATP synthase inhibitor oligomycin, the uncoupling agent FCCP (capable of dissipating the mitochondrial proton electrochemical gradient) and the electron transport inhibitors rotenone (complex I inhibitor) and antimycin A (complex III inhibitor) allow multiple parameters to be determined in a single experiment (Figure 3A).

Basal respiration is strongly influenced by the presence of different substrates in the incubation medium, membrane leak, and by ATP turnover [41]. MiR05 medium contains abundant phosphate, but no other carbon energy substrates; the non-reducing sugar sucrose is cell impermeable and not a substrate for HeLa cell metabolism [42]. Basal HeLa cell respiration (Figure 3Ba) under these conditions (utilizing endogenous intracellular substrates/stores) was higher in queuine deficient relative to queuine supplemented cells, though not statistically significant (Figure 3C). With the addition of oligomycin, the entire cellular ATP production is shifted to glycolysis, with a concomitant drop in the rate of oxygen consumption, reflecting a decrease in electron flow through the ETC under these conditions (Figure 3Bb). This state reveals the leak rate of respiration, and shows that queuine deficient cells have an increased loss of proton-motive force back through the inner mitochondrial membrane (Figure 3D), at least partially accounting for the increased basal respiration seen in these cells. The rate of mitochondrial ATP turnover can be estimated from the difference in respiration between basal rate and the oligomycin inhibited rate (Figure 3Bc). The data shows that queuine deficient cells have a higher rate of ATP turnover compared to queuine supplemented cells, but again the difference is not statistically significant (Figure 3E). 

The maximum respiration rate (Figure 3Bd) is induced by the addition of the uncoupling agent FCCP to reveal the maximal rate of substrate oxidation and electron transport by the mitochondria, whereas the spare respiratory capacity (Figure 3Ba–d) reports on the ability of a cell to respond to increased energy demand. The values obtained suggested that the mitochondria of queuine deficient cells have similar rates of maximal respiration to queuine supplemented cells (Figure 3F) and do not display an enhanced ability to respond to energy requirements (Figure 3G). The differences in proton leak determined above, question whether the coupling state of the mitochondria—the proportion of protons used for mitochondrial ATP production relative to the leak—may be affected by queuine [41,43]. However, coupling efficiency (c/a in Figure 3B) did not differ in the absence or presence of queuine. On the contrary, a significant reduction (*p* < 0.05) in the respiratory control ratio (RCR) was observed in queuine deficient cells, indicating that the mitochondria have a reduced capacity to oxidize respiratory substrates relative to proton leak (Figure 3Bb,d; state 3u/state 4o), i.e., increased proton leak in the presence of oligomycin (the denominator in the calculation) disproportionally accounts for oxygen consumption by the ETC when queuine is removed from the cells. 

In HeLa cells in culture, glucose metabolism is highly skewed towards lactate production with only minor amounts entering the citric acid cycle (estimated at 4–5%). Instead, glutamine contributes up to 65% of energy supply in the presence of glucose [44]. In this context of a prominent glycolytic dependence, any potential effects of queuine on mitochondrial function could be relatively moderate and require careful scrutiny. Furthermore, the mitochondrial genome encodes multiple subunits of the ETC for complexes I, III, IV, and V, the translation of which could potentially be impacted by the absence of Q-modification in tRNA_GUN_. Therefore, the electron carrying complexes of the ETC were evaluated in isolation by measuring changes in oxygen consumption in response to added substrates and inhibitors (Figure 3A and I). Since few substrates/inhibitors can enter the cell directly, digitonin was used to selectively permeabilize the plasma membrane by cholesterol precipitation. Complex I was examined by the addition of the NADH-linked substrates glutamate and malate together with an excess of ADP before being inhibited with rotenone. The FADH-linked substrate succinate was used to feed electrons into complex II before being inhibited by malonate. Complex III activity was examined using glycerol-3-phosphate (a substrate for the mitochondrial glycerol-3-phosphate dehydrogenase enzyme) and subsequently inhibited with antimycin A. The oxygen consumption of complex IV was assessed by the addition of ascorbate and TMPD, the latter being an artificial electron donor which can feed electrons directly to cytochrome c from ascorbate. Finally, the addition of potassium cyanide was used to inhibit all mitochondrial-dependent oxygen consumption in the cell, demonstrating that the autoxidation of TMPD is not contributing to oxygen consumption. From the representative trace (upper panel) and averaged graphed data (lower panel) it is apparent that queuine has no statistically significant effect upon the activity of individual complexes of the ETC.

Lastly, in permeabilized cells, the ATP/O ratio was ascertained, providing data on the stoichiometric efficiency of oxidative phosphorylation (i.e., ATP produced per oxygen consumed), with a representative trace shown (Figure 3J). This ratio was determined by the amount of oxygen consumed in permeabilized cells provided with glutamate/malate as a source of reducing equivalents when a limiting, defined amount of ADP was added (50 µM). However, no difference in the ATP/O ratio (State 3/State 4) was observed between queuine containing or queuine lacking cells (Figure 3K). Unexpectedly however—given the lack of any difference in ATP/O ratio—the rate at which oxygen consumption occurred, and hence the operating rate of the F_1_F_O_-ATP synthase was found to be lower in queuine deficient cells (Figure 3L). Indeed, the level of cellular ATP (evaluated using CellTiter-Glo reagent) was significantly reduced in queuine deficient cells relative to queuine supplemented cells (Figure 3M). 

### 3.4. Queuine Deficiency Permits the F_1_F_0_ ATP Synthase to Operate in the Reverse Mode

Our earlier observations showing queuine deficiency is associated with an increased proton leak, a decreased rate of ATP synthesis (forward reaction of the F_1_F_O_ ATP synthase), and a reduction in cellular ATP levels indicate mitochondrial dysfunction in the absence of queuine. An inability to fulfill the ATP requirements of the cell from mitochondrial respiration would necessarily force an increased rate of aerobic glycolysis to meet the resulting energy shortfall. To accentuate the activity (and hence dysfunction) of the mitochondria, HeLa cells were grown in culture medium in which glucose was substituted with galactose. The metabolism of galactose through the glycolytic pathway provides no net yield of ATP, and has been shown to induce a higher rate of mitochondrial respiration in HeLa cells [39].

**Figure 4:**
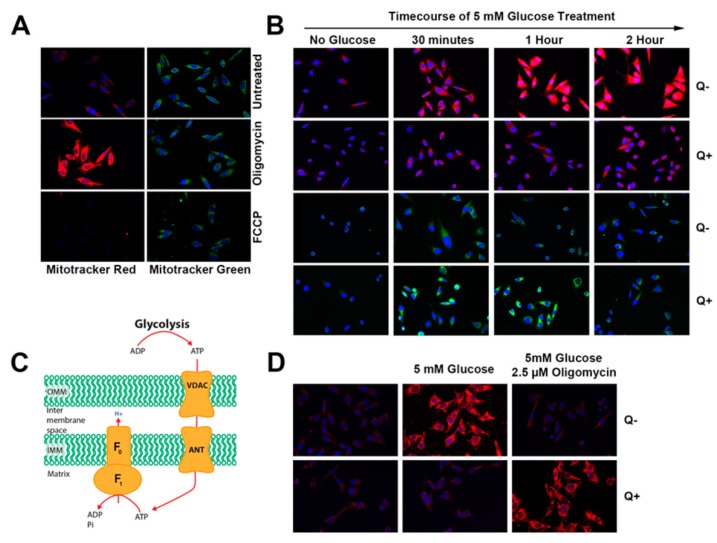
The F_1_F_O_ ATP synthase operates in reverse under queuine deficient conditions. (**A**) Cells were cultured in DMEM-FBS medium on cover slips, treated with 2.5 µM oligomycin or 5 µM FCCP and concurrently stained with Mitotracker Red CMXRos (50 nM) and Mitotracker Green (40 nM) before being fixed, permeabilized, and stained with DAPI dye. (**B**) Galactose adapted HeLa cells, cultured in the absence or presence of 1 µM queuine, were administered 5 mM glucose for the time durations shown, and concurrently with Mitotracker dye (for the last 15 min) prior to fixation. (**C**) The ATP synthase (complex V) consists of two functional domains, F_1_ and F_O_. The F_O_ domain facilitates the passage of protons from the intermembrane space to the matrix transferring the protomotive energy to F_1_, where ADP is phosphorylated to ATP. Normal respiring mitochondria favor ATP synthesis due to a high membrane potential, however, when the potential falls below a threshold, the F_1_F_O_ ATP synthase can operate in reverse to hydrolyse ATP and pump protons into the intermembrane space. (**D**) Galactose adapted HeLa cells were cultured in the absence or presence of 1 µM queuine. Glucose (5 mM) was administered to the cells for 15 min and subsequently, oligomycin (2.5 μM) and Mitotracker RedCMXRos were added for a further 15 min before fixation and imaging.

Fluorescent dyes allow a qualitative analysis of the mitochondrial membrane potential (Δ*ψ*_m_) in intact cells at single cell resolution and allow an evaluation of mitochondrial bioenergetics under physiological (unperturbed) conditions. Previously, it has been shown that the fluorescence of CMXRosamine is dependent on Δ*ψ*_m_ whereas the accumulation of the MitoTracker Green (MTG) probe is not related to Δ*ψ*_m_ and instead may be used as a qualitative tool for determining mitochondrial volume [45]. Therefore, we chose to use Mitotracker Red CMXRos as an indicator of membrane potential, titrating the concentration in preliminary studies to ensure operation in a ‘non-quench mode’ [41]. In validating the conditions of our study, HeLa cells were grown in DMEM-FBS medium (queuine containing) and either left untreated or administered oligomycin or FCCP followed by staining with CMXRos and MTG (Figure 4A). Oligomycin treated cells demonstrated greatly increased mitochondrial CMXRos fluorescence, whereas pre-treatment with FCCP fully dissipated fluorescence, as would be expected (left panels). By contrast, cells treated with MTG dye demonstrated no change in fluorescence across the three conditions (right panels).

Using the conditions above, we examined the consequence of providing the galactose-grown cells with a physiological concentration (5 mM) of glucose (Figure 4B). This situation is reflective of the natural in situ stress of hypoxia/reperfusion and substrate starvation/replenishment experienced by cancer cells due to changes in the tumor microenvironment. In queuine lacking cells, glucose addition led to a prominent increase in membrane potential, peaking at two hours (upper panel). By contrast, no such change in CMXRos was observed in queuine supplemented cells (upper-middle panel). The intensity of MTG remained unchanged in response to glucose (lower panels). Therefore, it appears that queuine deficiency can lead to substrate-dependent mitochondrial hyperpolarization. Studies have shown that mitochondria of cancer cells and cancer stem cells are hyperpolarized when compared to normal cells and that this relates to increased cancer invasiveness [46,47,48,49]. The exact cause of the hyperpolarization in cancer cells is not known, however it is suspected that reversal of the F_1_F_O_ ATP synthase (i.e., ATP hydrolysis) together with reversal of the adenine nucleotide transporters could account for the effect (Figure 4C), harvesting the ATP generated from glycolysis (2 ATP per glucose molecule) to sustain Δ*ψ*_m_ [50].

Therefore, in order to determine if the hyperpolarization observed in queuine free conditions arises from the hydrolysis of glucose-derived ATP by the F_1_F_O_ ATP synthase, HeLa cells that had been adapted to galactose medium were treated with glucose alone or treated concurrently with 5 mM glucose and 1 μg/mL oligomycin (Figure 4D). As observed previously, queuine deprived cells showed a marked increase in membrane potential when the medium was changed from 0 mM glucose (i.e., galactose containing medium; top left panel, Figure 4D) to medium supplemented with 5 mM glucose (top middle panel, Figure 4D). However, oligomycin inhibited this increase (top right panel, Figure 4D), which in normal functioning cells is the opposite of what would be expected i.e., inhibiting the F_1_F_O_ ATP synthase with oligomycin should increase Δ*ψ*_m_. Juxtaposing the observations made in queuine deficient cells, the cells to which queuine was administered showed no hyperpolarization in response to glucose (compare bottom left and middle panels) but did display increased membrane potential when treated with oligomycin (bottom right panel). We therefore conclude, that queuine deficiency leads to a drop in cellular ATP supply and that this manifests in the reversal of the F_1_F_O_ ATP synthase and adenine nucleotide transporter (ANT), biasing metabolism towards aerobic glycolysis.

## 4. Discussion

Numerous solid and circulatory cancers have shown decreased tRNA incorporation of the queuine micronutrient [6]. This deficiency correlates with the aggressiveness and stage of the tumor [11,12,14] and thus the absence of queuine may be likely to bestow a growth or survival advantage to the cancer cell. In this study, we demonstrate that queuine depletion induces a Warburg-type metabolism in human cervical HeLa cells (or more specifically, exacerbates what is already an existing Warburg phenotype) and can cause a hyperpolarization of the inner mitochondrial membrane—a trait commonly observed in cancer [50].

The increased proliferation of cancer cells depends upon a high rate of glycolysis for biosynthetic purposes, with pyruvate being diverted to lactate, rather than being directed to the TCA cycle. In many tumor types, glutamine deamination (producing ammonia and glutamate) is used to produce α-ketoglutarate to replenish the TCA cycle and provide intermediates for the synthesis of amino acids, nucleotides and lipids, with the production of ATP and lactate [51]. Queuine deficiency was observed to induce these prototypical metabolic changes, resulting in increased glycolysis and glutaminolysis and the production of lactate and ammonia. Similar to previous studies [17], an elevation in LDH activity was also observed in the absence of queuine. The importance of LDH and lactate induction to cancer progression is such that it has been proposed as an essential underlying purpose of the Warburg effect [52]. Therefore, the decreased Q-modification of tRNA is likely to be a deliberate and desirable objective of a developing tumor, to bring about a number of selective survival advantages that inter alia lead to an increased supply of bioenergetic intermediates, resistance to hypoxia and apoptosis, and an acidification of the microenvironment to suppress the function of infiltrating immune cells [53,54].

Otto Warburg proposed that the reliance of cancer cells on aerobic glycolysis stems from a defect in mitochondrial oxidative phosphorylation [55]. Although it is now clear that most cancer cells do not have a defect in mitochondrial metabolism, rare mutations in the TCA enzymes succinate dehydrogenase and fumarate hydratase have been shown to force a switch to Warburg metabolism and contribute to inherited and sporadic cancers [5]. As queuosine is one of only four post-translational modifications found in the wobble position of mitochondrial tRNA, we investigated how mitochondrial bioenergetics may be affected by its absence. In intact cells, queuine deficiency led to an increased rate of proton leak back across the inner mitochondrial membrane relative to queuine-supplemented cells. This is reflected in a reduced capacity to produce ATP from substrate oxidation, as revealed by a statistically significant decrease in the RCR. An increased membrane leak may also explain why the addition of oligomycin to queuine deficient cells did not result in an increased Δ*ψ*_m_ (TPMP accumulation), contrary to what was observed for queuine-supplemented cells. Despite numerous channel-forming proteins of the ETC being encoded by the mitochondria, queuine deficiency did not appear to affect the activity of the individual complexes (I-IV) when examined in permeabilized cells.

A slower forward rate of the F_1_F_O_-ATP synthase (determined in permeabilized cells) and a reduction of cellular ATP levels were observable under queuine deficient conditions, which is interesting from the perspective that two subunits of the ATP synthase (ATP6 and ATP8) are encoded in the mitochondrial genome. Therefore, we speculate that queuine may influence the activity of the ATP synthase or mitochondrial transport proteins, such as the adenine nucleotide transporter (ANT), which is responsible for importing cytosolic ADP into the mitochondrial matrix in exchange for ATP. Typically respiring mitochondria have a Δ*ψ*_m_ of between –150 and –180 mV along with a ∆pH of about 0.5–1.0, to give a total proton electrochemical gradient (Δµ_H+_), providing favorable conditions for ATP production by the F_1_F_O_-ATP synthase [56]. Although cancer cells frequently present with a hyperpolarized Δ*ψ*_m_ (in some cases as high as –210 mV), this is extremely suboptimal for ATP synthesis [50] (and references 8-17 therein). By forcing HeLa cells to grow on galactose medium we could show that glucose replenishment (i.e., re-activating aerobic glycolysis) leads to mitochondrial hyperpolarization only in queuine deficient cells. Furthermore, our data indicated that glycolysis was the source of the ATP used to hyperpolarize the membrane. Since studies show that tumor aggressiveness is promoted by a mitochondrial hyperpolarization [46,47], this represents another example of how cancer cells would benefit from Q-hypomodification in tRNA.

While under conventional conditions, the mitochondrial F_1_F_O_-ATPase operates in the direction of ATP synthesis, it is only constrained to do so by continual regeneration of the Δµ_H+_ as a result of electron flow through the ETC and the cellular consumption of ATP. It has long been recognized that a mitochondrial Δµ_H+_ can be readily generated and maintained by the F_1_F_O_-ATPase at the expense of ATP derived from glycolysis [50]. Interestingly, under these conditions the ATP/ADP translocator and the phosphate/H^+^ symporter also operate in reverse direction to contribute to the gradient. This behavior was recognized as a bioenergetic feature of cancer cells and is readily observed following depolarization of the Δ*ψ*_m_ by F_1_F_O_-ATPase inhibition with oligomycin. By contrast, in cells using the F_1_F_O_-ATPase in the direction of ATP synthesis (i.e., in classical oxidative phosphorylation) the addition of oligomycin leads to a hyperpolarization of the potential [57,58]. These two opposing effects of oligomycin are precisely what were observed in the absence and presence of queuine.

The means by which Q-hypomodification is achieved in cancer is currently an open question. Disruption to queuine bioavailability, uptake, and salvage and a decrease in TGT activity (either expression or through physiological inhibitors) have been implicated [14,19,59,60] and evidence from cell lines confirms some of these possibilities [60]. In vivo tumor progression is driven by cell autonomous changes and a complex reciprocal (at times symbiotic) exchange between the cancer cell and its microenvironment [53]. In the case of solid tumors—developing at a distance from the vasculature—it is tempting to speculate that the reduced blood supply could affect queuine bioavailability and act as a proxy for reduced substrate supply. Contradicting this conjecture however is the fact that circulatory cancers also have low Q-tRNA levels and ostensibly have no limitation on queuine supply from the serum. It is noteworthy that Q-tRNA is also decreased under normal physiological circumstances. For example, the tRNA of pre-natal mice, young rats, regenerating liver, and rapidly dividing sheep reticulocytes all have been found to be Q-hypomodifed [61,62,63,64], suggesting the absence of queuine is concordant with and relevant to the replicative, undifferentiated cellular state.

In conclusion, the observed Q-hypomodification in cancer is expected to contribute to cancer cell survival and immune resistance through promotion of Warburg metabolism. Furthermore, the association of queuine deficiency with mitochondrial hyperpolarization could have important implications for the resistance of cancer cells to apoptotic cell death.

## Supplementary Materials

The following are available online at https://www.mdpi.com/2072-6643/12/3/871/s1, Figure S1: Queuine addition to serum-free medium results in full Q-modification of histidyl and asparaginyl tRNA in HeLa cells.

## References

[1] Zhu W.Q., Sun X., Xing L., Li M., Yue J., Qu W., Kong L., Yu J. (2012). Oesophageal squamous cell carcinoma: Relationship between fluorine-18 fludeoxyglucose positron emission tomography ct maximum standardised uptake value, metabolic tumour volume, and tumour, node and metastasis classification. Br. J. Radiol..

[2] Gatenby R.A., Gillies R.J. (2004). Why do cancers have high aerobic glycolysis?. Nat. Rev. Cancer.

[3] DeBerardinis R.J., Lum J.J., Hatzivassiliou G., Thompson C.B. (2008). The biology of cancer: Metabolic reprogramming fuels cell growth and proliferation. Cell Metab..

[4] Vander Heiden M.G., Cantley L.C., Thompson C.B. (2009). Understanding the warburg effect: The metabolic requirements of cell proliferation. Science.

[5] Levine A.J., Puzio-Kuter A.M. (2010). The control of the metabolic switch in cancers by oncogenes and tumor suppressor genes. Science.

[6] Fergus C., Barnes D., Alqasem M.A., Kelly V.P. (2015). The queuine micronutrient: Charting a course from microbe to man. Nutrients.

[7] Harada F., Nishimura S. (1972). Possible anticodon sequences of tRNA His, tRNA Asm, and tRNA Asp from Escherichia coli B. Universal presence of nucleoside Q in the first postion of the anticondons of these transfer ribonucleic acids. Biochemistry.

[8] Randerath E., Agrawal H.P., Randerath K. (1984). Specific lack of the hypermodified nucleoside, queuosine, in hepatoma mitochondrial aspartate transfer RNA and its possible biological significance. Cancer Res..

[9] Morl M., Dorner M., Paabo S. (1995). C to U editing and modifications during the maturation of the mitochondrial tRNA(Asp) in marsupials. Nucleic Acids Res..

[10] Suzuki T. (2014). A complete landscape of post-transcriptional modifications in mammalian mitochondrial tRNAs. Nucleic Acids Res..

[11] Baranowski W., Dirheimer G., Jakowicki J.A., Keith G. (1994). Deficiency of queuine, a highly modified purine base, in transfer RNAs from primary and metastatic ovarian malignant tumors in women. Cancer Res..

[12] Huang B.S., Wu R.T., Chien K.Y. (1992). Relationship of the queuine content of transfer ribonucleic acids to histopathological grading and survival in human lung cancer. Cancer Res..

[13] Emmerich B., Zubrod E., Weber H., Maubach P.A., Kersten H., Kersten W. (1985). Relationship of queuine-lacking transfer RNA to the grade of malignancy in human leukemias and lymphomas. Cancer Res..

[14] Dirheimer G., Baranowski W., Keith G. (1995). Variations in trna modifications, particularly of their queuine content in higher eukaryotes. Its relation to malignancy grading. Biochimie.

[15] Aytac U., Gunduz U. (1994). Q-modification of tRNAs in human brain tumors. Cancer Biochem. Biophys..

[16] Langgut W., Kersten H. (1990). The deazaguanine-derivative, queuine, affects cell proliferation, protein phosphorylation and the expression of the proto oncogenes c-fos and c-myc in HeLa cells. FEBS Lett..

[17] Reisser T., Langgut W., Kersten H. (1994). The nutrient factor queuine protects HeLa cells from hypoxic stress and improves metabolic adaptation to oxygen availability. Eur. J. Biochem./FEBS.

[18] Langgut W., Reisser T., Kersten H., Nishimura S. (1993). Modulation of epidermal growth factor receptor activity and related responses by the 7-deazaguanine derivative, queuine. Oncogene.

[19] Pathak C., Vinayak M. (2005). Modulation of lactate dehydrogenase isozymes by modified base queuine. Mol. Biol. Rep..

[20] Shindo-Okada N., Terada M., Nishimura S. (1981). Changes in amount of hypo-modified tRNA having guanine in place of queuine during erythroid differentiation of murine erythroleukemia cells. Eur. J. Biochem. / FEBS.

[21] Chen Y.L., Wu R.T. (1994). Altered queuine modification of transfer RNA involved in the differentiation of human K562 erythroleukemia cells in the presence of distinct differentiation inducers. Cancer Res..

[22] Morgan C.J., Merrill F.L., Trewyn R.W. (1996). Defective transfer RNA-queuine modification in C3H10T1/2 murine fibroblasts transfected with oncogenic ras. Cancer Res..

[23] Muralidhar G., Trewyn R.W. (1987). Enhancement of the chemical transformation of chinese hamster embryo cells in vitro by 7-methylguanine. Cancer Res..

[24] Muralidhar G., Ochieng J., Trewyn R.W. (1989). Altered queuine modification of transfer RNA involved in the in vitro transformation of chinese hamster embryo cells. Cancer Res..

[25] Ishiguro K., Sartorelli A.C. (1985). Enhancement of the differentiation-inducing properties of 6-thioguanine by hypoxanthine and its nucleosides in HL-60 promyelocytic leukemia cells. Cancer Res..

[26] Kretz K.A., Katze J.R., Trewyn R.W. (1987). Guanine analog-induced differentiation of human promyelocytic leukemia cells and changes in queuine modification of tRNA. Mol. Cell. Biol..

[27] French B.T., Patrick D.E., Grever M.R., Trewyn R.W. (1991). Queuine, a tRNA anticodon wobble base, maintains the proliferative and pluripotent potential of HL-60 cells in the presence of the differentiating agent 6-thioguanine. Proc. Natl. Acad. Sci. USA.

[28] Morgan C.J., Chawdry R.N., Smith A.R., Siravo-Sagraves G., Trewyn R.W. (1994). 6-thioguanine-induced growth arrest in 6-mercaptopurine-resistant human leukemia cells. Cancer Res..

[29] Zaborske J.M., Bauer DuMont V.L., Wallace E.W.J., Pan T., Aquadro C.F., Drummond D.A. (2014). A Nutrient-Driven tRNA Modification Alters Translational Fidelity and Genome-wide Protein Coding across an Animal Genus. PLoS Biol..

[30] Hyslop S.J., James A.M., Maw M., Fischel-Ghodsian N., Murphy M.P. (1997). The effect on mitochondrial function of the tRNA Ser(UCN)/COI A7445G mtDNA point mutation associated with maternally-inherited sensorineural deafness. Biochem. Mol. Biol. Int..

[31] Appleby R.D., Porteous W.K., Hughes G., James A.M., Shannon D., Wei Y.H., Murphy M.P. (1999). Quantitation and origin of the mitochondrial membrane potential in human cells lacking mitochondrial DNA. Eur. J. Biochem. / FEBS.

[32] Langgut W. (1993). Changes of the phosphorylation of membrane-associated proteins following treatment of HeLa cells with the guanine analogue, queuine. Biofactors.

[33] Rakovich T., Boland C., Bernstein I., Chikwana V.M., Iwata-Reuyl D., Kelly V.P. (2011). Queuosine deficiency in eukaryotes compromises tyrosine production through increased tetrahydrobiopterin oxidation. J. Biol. Chem..

[34] Miao P., Sheng S., Sun X., Liu J., Huang G. (2013). Lactate dehydrogenase a in cancer: A promising target for diagnosis and therapy. IUBMB Life.

[35] Page B., Page M., Noel C. (1993). A new fluorometric assay for cytotoxicity measurements in-vitro. Int. J. Oncol..

[36] Rampersad S.N. (2012). Multiple applications of alamar blue as an indicator of metabolic function and cellular health in cell viability bioassays. Sensors (Basel).

[37] Trounce I.A., Kim Y.L., Jun A.S., Wallace D.C. (1996). Assessment of mitochondrial oxidative phosphorylation in patient muscle biopsies, lymphoblasts, and transmitochondrial cell lines. Methods Enzymol..

[38] Alirol E., Martinou J.C. (2006). Mitochondria and cancer: Is there a morphological connection?. Oncogene.

[39] Rossignol R., Gilkerson R., Aggeler R., Yamagata K., Remington S.J., Capaldi R.A. (2004). Energy substrate modulates mitochondrial structure and oxidative capacity in cancer cells. Cancer Res..

[40] Ross M.F., Prime T.A., Abakumova I., James A.M., Porteous C.M., Smith R.A., Murphy M.P. (2008). Rapid and extensive uptake and activation of hydrophobic triphenylphosphonium cations within cells. Biochem. J..

[41] Brand M.D., Nicholls D.G. (2011). Assessing mitochondrial dysfunction in cells. Biochem. J..

[42] Eagle H., Barban S., Levy M., Schulze H.O. (1958). The utilization of carbohydrates by human cell cultures. J. Biol. Chem..

[43] Hall A., Larsen A.K., Parhamifar L., Meyle K.D., Wu L.P., Moghimi S.M. (2013). High resolution respirometry analysis of polyethylenimine-mediated mitochondrial energy crisis and cellular stress: Mitochondrial proton leak and inhibition of the electron transport system. Biochim. Biophys. Acta.

[44] Reitzer L.J., Wice B.M., Kennell D. (1979). Evidence that glutamine, not sugar, is the major energy source for cultured HeLa cells. J. Biol. Chem..

[45] Pendergrass W., Wolf N., Poot M. (2004). Efficacy of MitoTracker Green and CMXrosamine to measure changes in mitochondrial membrane potentials in living cells and tissues. Cytom. Part A J. Int. Soc. Anal. Cytol..

[46] Heerdt B.G., Houston M.A., Augenlicht L.H. (2005). The intrinsic mitochondrial membrane potential of colonic carcinoma cells is linked to the probability of tumor progression. Cancer Res..

[47] Heerdt B.G., Houston M.A., Augenlicht L.H. (2006). Growth properties of colonic tumor cells are a function of the intrinsic mitochondrial membrane potential. Cancer Res..

[48] Wang J., Shi X., Johnson R.H., Kelbauskas L., Zhang W., Meldrum D.R. (2013). Single-cell analysis reveals early manifestation of cancerous phenotype in pre-malignant esophageal cells. PLoS ONE.

[49] Duan J.J., Qiu W., Xu S.L., Wang B., Ye X.Z., Ping Y.F., Zhang X., Bian X.W., Yu S.C. (2013). Strategies for isolating and enriching cancer stem cells: Well begun is half done. Stem Cells Dev..

[50] Forrest M.D. (2015). Why Cancer Cells Have a More Hyperpolarised Mitochondrial Membrane Potential and Emergent Prospects for Therapy. http://biorxiv.org/content/early/2015/08/21/025197.

[51] Cetinbas N.M., Sudderth J., Harris R.C., Cebeci A., Negri G.L., Yilmaz O.H., DeBerardinis R.J., Sorensen P.H. (2016). Glucose-dependent anaplerosis in cancer cells is required for cellular redox balance in the absence of glutamine. Sci. Rep..

[52] San-Millan I., Brooks G.A. (2016). Reexamining cancer metabolism: Lactate production for carcinogenesis could be the purpose and explanation of the warburg effect. Carcinogenesis.

[53] Lehuede C., Dupuy F., Rabinovitch R., Jones R.G., Siegel P.M. (2016). Metabolic plasticity as a determinant of tumor growth and metastasis. Cancer Res..

[54] Molon B., Cali B., Viola A. (2016). T cells and cancer: How metabolism shapes immunity. Front. Immunol..

[55] Warburg O. (1956). On the origin of cancer cells. Science.

[56] Campanella M., Parker N., Tan C.H., Hall A.M., Duchen M.R. (2009). IF(1): Setting the pace of the F(1)F(o)-ATP synthase. Trends Biochem. Sci..

[57] Akerman K.E. (1979). Qualitative measurements of the mitochondrial membrane potential in situ in Ehrlich ascites tumour cells using the safranine method. Biochim Biophys Acta.

[58] Nolan D.P., Voorheis H.P. (1992). The mitochondrion in Trypanosoma brucei is energised by the electrogenic translocation of H+ catalysed by the F1F0-ATPase. Eur. J. Biochem..

[59] Jacobson K.B., Farkas W.R., Katze J.R. (1981). Presence of queuine in drosophila melanogaster: Correlation of free pool with queuosine content of tRNA and effect of mutations in pteridine metabolism. Nucleic Acids Res..

[60] Morris R.C., Galicia M.C., Clase K.L., Elliott M.S. (1999). Determination of queuosine modification system deficiencies in cultured human cells. Mol. Genet. Metab..

[61] Singhal R.P., Kopper R.A., Nishimura S., Shindo-Okada N. (1981). Modification of guanine to queuine in transfer RNAs during development and aging. Biochem. Biophys. Res. Commun..

[62] Frazer J.M., Yang W.K. (1972). Isoaccepting transfer ribonucleic acids in liver and brain of young and old BC3F 1 mice. Arch. Biochem. Biophys..

[63] Costa A., Pais de Barros J.P., Keith G., Baranowski W., Desgres J. (2004). Determination of queuosine derivatives by reverse-phase liquid chromatography for the hypomodification study of Q-bearing tRNAs from various mammal liver cells. J. Chromatogr. BAnal. Technol. Biomed. Life Sci..

[64] Landin R.M., Boisnard M., Petrissant G. (1979). Correlation between the presence of tRNA His GUG and the erythropoietic function in foetal sheep liver. Nucleic Acids Res..

