# Supplementary material for: Queuine Micronutrient Deficiency Promotes Warburg Metabolism and Reversal of the Mitochondrial ATP Synthase in Hela Cells
Source: Nutrients. 2020 Mar 24;12(3):871. doi: 10.3390/nu12030871 (PMC7146442; doi:10.3390/nu12030871)
Supplement: Supplementary file 1 [file nutrients-12-00871-s001.pdf]

Nutrients **2020**, *12*, 871; doi:10.3390/nu12030871

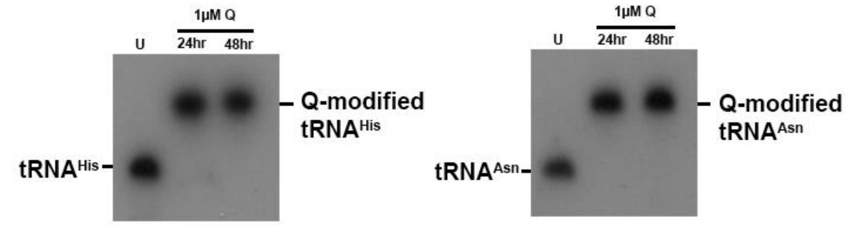

Figure S1. *Queuine addition to serum-free medium results in full Q-modification of histidyl and asparaginyl tRNA in HeLa cells.* Cells were seeded at 3 x 10^4 per cm^2 in serum free medium (queuine deficient) for 5 days. They were then either supplemented with 1 µM queuine base and incubated for 24 or 48 hours, or left untreated (U). RNA was isolated and the tRNA modification status determined by electrophoretic separation on APB gels, followed by Northern blotting. The tRNA signals were detected using ^{32}P-labelled oligonucleotide probes specific for $tRNA^{His}$ or $tRNA^{Asn}$.
